# Genotype Diversity of Newcastle Disease Virus in Nigeria: Disease Control Challenges and Future Outlook

**DOI:** 10.1155/2018/6097291

**Published:** 2018-12-02

**Authors:** Muhammad Bashir Bello, Khatijah Mohd Yusoff, Aini Ideris, Mohd Hair-Bejo, Ben P. H. Peeters, Abdurrahman Hassan Jibril, Farouk Muhammad Tambuwal, Abdul Rahman Omar

**Affiliations:** ^1^Faculty of Veterinary Medicine, Usmanu Danfodiyo University, PMB 2346, Sokoto, Nigeria; ^2^Laboratory of Vaccines and Immunotherapeutics, Institute of Bioscience, Universiti Putra Malaysia, 43400 Serdang, Selangor, Malaysia; ^3^Faculty of Biotechnology and Biomolecular Sciences, Universiti Putra Malaysia, 43400, Selangor, Malaysia; ^4^Faculty of Veterinary Medicine, Universiti Putra Malaysia, 43400 Serdang, Selangor, Malaysia; ^5^Division of Virology, Central Veterinary Institute, Wageningen University, P.O. Box 65, NL8200 Lelystad, Netherlands

## Abstract

Newcastle disease (ND) is one of the most important avian diseases with considerable threat to the productivity of poultry all over the world. The disease is associated with severe respiratory, gastrointestinal, and neurological lesions in chicken leading to high mortality and several other production related losses. The aetiology of the disease is an avian paramyxovirus type-1 or Newcastle disease virus (NDV), whose isolates are serologically grouped into a single serotype but genetically classified into a total of 19 genotypes, owing to the continuous emergence and evolution of the virus. In Nigeria, molecular characterization of NDV is generally very scanty and majorly focuses on the amplification of the partial F gene for genotype assignment. However, with the introduction of the most objective NDV genotyping criteria which utilize complete fusion protein coding sequences in phylogenetic taxonomy, the enormous genetic diversity of the virus in Nigeria became very conspicuous. In this review, we examine the current ecological distribution of various NDV genotypes in Nigeria based on the available complete fusion protein nucleotide sequences (1662 bp) in the NCBI database. We then discuss the challenges of ND control as a result of the wide genetic distance between the currently circulating NDV isolates and the commonest vaccines used to combat the disease in the country. Finally, we suggest future directions in the war against the economically devastating ND in Nigeria.

## 1. Introduction

Poultry production is globally threatened by a highly devastating disease of birds called Newcastle disease (ND). The disease was named after a place known as Newcastle Upon Tyne, in England where it was reported for the first time in 1926 [1]. The disease was also reported around the same time in Java, Indonesia [2]. Amazingly, its geographic distribution slowly expanded, leading to a well-established pandemic of the disease barely two decades after its novel emergence [3]. Subsequently in the late 1960s, the second pandemic of the disease occurred with an incredibly high speed, taking only four years to spread throughout the world, probably due to extensive commercialization of poultry production and the improvement of air transport systems which facilitated the exchange of exotic birds into new areas [4]. Although this pandemic was quickly placed under control with the then available ND vaccines, the third pandemic still occurred around the early 1980s among the racing pigeons [5, 6]. This particular pandemic proved to be difficult to control because of the nature of racing pigeon husbandry system. Eventually, the pandemic virus spilt over to the domesticated chicken and caused serious economic losses in the poultry subsector [7]. The fourth pandemic, which started around the mid-1980s in the South-Eastern Asia, is currently believed to be on-going and has so far spread extensively to the Middle East, Europe, America, and Africa [8–11]. In Nigeria, the first official documentation of ND was in 1952 (Hill et al. 1953) and at present, the disease has been reported in all the ago-ecological zones of the country [12–15].

The aetiology of ND is an avian paramyxovirus type-1, which is a member of the genus* Avulavirus *in the family Paramyxoviridae [16]. The genetic material of the virus is a negative sense RNA made up of six genes encoding six structural proteins in the order 3′NP-P-M-F-HN-L5′ [17, 18]. Pathogenicity indices such as the mean death time (MDT) in 9-10-day-old embryonated chicken eggs and the intracerebral pathogenicity index (ICPI) in 1-day-old chicks are often used to classify the virus isolates into velogenic, mesogenic, and lentogenic strains [19]. The velogenic strains (neurotrophic or viscerotropic) are highly fatal and therefore demonstrate the severest clinical form of the disease, causing haemorrhagic gastroenteritis, pneumonia, and/or encephalitis [20, 21]. The mesogenic strains which are moderately pathogenic cause respiratory and neurological symptoms but with significantly low mortality [22, 23]. On the other hand, the lentogenic pathotypes are of extremely low virulence, causing only mild respiratory or asymptomatic enteric disease in the affected chicken [23, 24]. Interestingly, the major determinant of NDV virulence has been traced to be the amino acid composition of its fusion protein cleavage site [25]. All virulent strains have multiple basic amino acid residues at positions 112-116 and a phenyl alanine at position 117, making them cleavable by most of the ubiquitously distributed intracellular furin-like proteases in various chicken tissues [26]. In contrast, the F cleavage site of the avirulent strains is normally composed of monobasic amino acid residues at positions 112-116 and a leucine residue at position 117 [27]. Thus the chemistry of Fo cleavage site can be used as a good index for rapid pathotyping of NDV using molecular based assays.

In Nigeria, molecular characterization of ND outbreaks was until recently very scanty and largely focused on the partial F gene sequences for phylogenetic grouping of isolates [29, 30]. With the introduction of more objective criteria that utilizes the complete F gene coding sequences for assigning new genotypes [31], the genetic diversity of NDV in Nigeria has become more apparent [32–34]. Unfortunately to date, the consequences of this genetic diversity on disease control using the available vaccines in the country remain poorly addressed. Therefore in this review, we analyze the current ecological distribution of NDV genotypes in various parts of Nigeria and discuss the implication of the genotype mismatch between the circulating field strains and the vaccine strains to ND control in the country.

## 2. Taxonomy and Global Distribution of Newcastle Disease Virus Isolates

Although all NDV strains are classified under one serotype [35], their genetic diversity is enormous [36–39]. In the past, various schemes have been concurrently used to classify NDV based on their genetic information. The first classification system proposed by the Aldous group divides all the isolates into six lineages and 13 sublineages [40]. An additional lineage and seven more sublineages were later proposed [41, 42]. The other scheme of NDV taxonomy proposed by Ballagi-Pordány et al. [3] and later substantiated by Czeglédi et al. [43] groups the NDV isolates into various genotypes and subgenotypes. Conflicts and confusion generated by these schemes of classification necessitated the need to develop unified criteria for NDV taxonomy. After analyzing the two systems extensively, Diel et al. [31] proposed the adoption of the genotype based classification not only because it is the most widely used, but also because it gives a stronger correlation between the intergenetic groups evolutionary distances and their phylogenetic relationships. Therefore, a unified nomenclature system was proposed for the then existing isolates and more comprehensive criteria for the assignment of newly emerged genotypes were proposed [31]. According to the criteria, classification of a new genotype will be based on the phylogenetic topology using the complete, not partial F gene coding sequences. Furthermore, at least four isolates obtained from epidemiologically distinct events must form a phylogenetic cluster with a bootstrap value of nothing less than 60%. In addition, the isolates should have an average interpopulation evolutionary distance of ≥ 10. However, a mean evolutionary distance of 3-10% shall be used to designate a new subgenotype within a group [31].

Using these objective criteria, NDV isolates have been broadly classified into class I and class II [44–46]. The class I isolates are all grouped into a single genotype and three subgenotypes because of their high genetic relatedness which is nearly 96% [47]. They are mostly isolated from wild and domesticated birds found in Africa, Asia, Europe, and America [36, 48, 49]. With the exception of one isolate that caused serious disease outbreak in the Northern Ireland around the early 1990s [50], all members of this class are considered of low virulence in chicken. On the contrary, the class II isolates are a mixture of viruses with diverse virulence potentials ranging from the most popular vaccine strains used for disease control to the highly virulent strains that cause outbreaks in different parts of the world (Table 1). According to the recent literatures, class II isolates are classified into genotypes I-XVIII, with majority of the genotypes being further subdivided into various subgenotypes [51–53]. For instance, genotype I isolates which are globally distributed are composed of three subgenotypes: 1a, 1b, and 1c most of which are considered lentogenic. Indeed, the widely reported Queensland V4 and Ulster/chicken/Ireland/1967 vaccine strains are all grouped under this genotype [52, 53]. However, Gould et al. [54] reported the occurrence of virulent genotype I isolates in Australia. Similarly, the genotype II isolates are a mixture of velogenic [37, 38] and lentogenic viruses such as LaSota and B1 strains used globally for disease control [55]. Isolates in this genotype have been majorly recovered from domestic fowl, chicken, and wild birds found in North and South America, Africa, Asia, and Europe [44, 45].

Isolates belonging to genotypes III, IV, V, and VI are all predicted or pathotyped to be virulent in chicken. The genotype III isolates which include the popular mesogenic Mukteshwar strain used as a vaccine strain were recovered from birds in Japan as early as 1930s and also in Pakistan around the mid 1970s before they subsequently resurge in China less than two decades ago [56, 57]. Likewise, the genotype IV isolates occurred among the European poultry before the 1940s and include the extensively characterized Herts/33 strain [3, 58]. However, isolates in this genotype are currently thought to be extinct [44, 45] due to the absence of their recent genetic information in the GenBank database. As for the genotype V isolates that emerged for the first time around the 1970s in America and spread to the European continent in 1980s [43, 59], their recovery from poultry has recently been reported in East Africa, suggesting the expansion of their geographic distribution [60] and their continuous evolution. So far, isolates in this genotype are divided into four distinct subgenotypes (Va, Vb, Vc, and Vd) because of their within-the-group heterogeneity. However, the genotype VI isolates which are cosmopolitan in distribution are much more heterogeneous genetically. They are currently divided into subgenotypes VIa-k [37, 38, 51] because of their enormous genetic diversity. In addition, they are mainly found in wild birds, chicken, and more frequently in domestic pigeons, hence the name pigeon paramyxoviruses [6, 52, 61].

Genotype VII isolates are arguably the most important group of NDV reported in the 21st century. From the year 2000 to date, these viruses have been incriminated in several economically important disease outbreaks in Asia, the Middle East, and some parts of America and South Africa [62–65]. Because of their extensive genetic diversity and continuous emergence, they are currently grouped into twelve subgenotypes (VIIa-l) [66] and are believed to be associated with the ongoing fourth pandemic of the disease. As a matter of fact, some of these subgenotypes are predicted to be the potential fifth ND pandemic viruses because of the recent expansion of their host range and geographic distribution as well as their increased virulence among the vaccinated birds [67–70]. In particular, subgenotype VIIi isolates have recently replaced the predominant VIIa isolates in countries such as Pakistan since 2011 [71]. Similarly, subgenotype VIIj isolates believed to have emerged from viruses circulating in China and Ukraine are increasingly isolated in several countries including Iran [72]. This complex genetic diversity of genotype VII NDV highlights the need to monitor the epidemiological dynamics of the emerging viruses so that effective vaccination program can be designed. Unlike the genotype VII isolates, members of genotype VIII taxon are less diverse both genetically and in terms of spatial distribution. Apart from the report on their occurrence in Malaysia, Singapore, China, Turkey, Argentina, and South Africa between the 1960s and 1990s [8, 17], no report exists on their emergence in other parts of the world in the recent times. Hence they are thought to currently cease circulation in domestic birds. In contrast, the genotype IX isolates are still evolving in wild birds and domesticated poultry since survey of NDV between 2008 and 2011 revealed their presence in China [73, 74]. Nevertheless, they are still considered to be among the early genotypes, having been isolated as early as 1940s [3]. However, unlike members of this genotype (genotype IX) which are mostly virulent in chicken, genotype X isolates are all predicted to be in the lentogenic class. Despite their restricted geographic distribution, they are still maintained between the turkeys and wild birds in Argentina and the United States of America [75, 76]. They are however among the less genetically diverse groups of NDV.

Perhaps the most geographically restricted group of NDV are the genotype XI isolates. They have only been reported from Madagascar, where they are believed to circulate between the wild birds and domestic chicken [35, 58]. Although they are all predicted to be virulent based on the chemistry of their F cleavage site, there are reports of their isolation from apparently normal unvaccinated birds in Madagascar [35]. Meanwhile the genotype XII isolates, which are all predicted to be virulent, have been reported from both China and America in geese and chicken, respectively [44, 45, 77]. The epidemiological connection between the isolates in America and those in China is however still not clear, since migratory birds have not so far been incriminated in carrying these viruses [31, 44, 45]. Genotype XIII isolates which have been recovered from birds in Europe, Asia, and Africa are all predicted to be virulent based on the amino acid composition of their F cleavage site [78]. They are thought to be continuously evolving especially in Asia and the Middle East. Currently, they are divided into subgenotypes XIIIa, XIIIb, and XIIIc [79, 80].

The rest of the NDV genotypes are all predicted to be virulent in chicken. Isolates belonging to genotypes XIV, XVII, and XVIII have been recovered mainly from domesticated birds such as chicken, turkeys, and guinea fowls. Each of these genotypes is currently divided into two subgenotypes, a and b [53]. Because their geographic distribution is restricted to the west and central Africa, they are often referred to as regional NDV genotypes. On the other hand, members of the genotype XV group are considered to be recombinant isolates that might have emerged from the suboptimally vaccinated poultry in China some two decades ago [44, 45]. However, it is doubtful if they are still maintained in the poultry due to the absence of report on their occurrence in the last 15 years. Finally, genotype XVI isolates which were isolated from the Mexican chicken as early the 1940s [81] are believed to have been maintained in either the vaccinated or wild birds unnoticed for quite several years. They were also isolated in the Caribbean islands between 1986 and 2008 [43].

## 3. Ecology of NDV Genotypes in Nigeria

Analysis of the complete F gene coding sequences (1662bp) for Nigerian strains of NDV available in the NCBI database reveals the occurrence of genetically distinct strains in various species of birds across the lengths and breadths of Nigeria (Table 2). Based on phylogenetic relationships and evolutionary distances, those isolates were grouped into class II genotypes I, VI, XIV, XVII, and XVIII. Except the genotype I isolates with GRQGRL amino acid motifs at positions 112-117 of the F gene, all other isolates considered in this study are predicted to be virulent in chicken based on the presence of multiple basic amino acid residues in their F cleavage sites (Table 1). Notably, among those virulent cleavage sites, the “RRQKRF” is the most diverse, being possessed by all the analyzed sequences except those from genotypes I and VIh. Furthermore, some strains from subgenotypes XVIIa, XIVb, and VIh display “RRRKRF” at their cleavage sites whereas only one isolate from subgenotype VIg, another one from subgenotype XVIIb, and four isolates from subgenotype VIh possess “KRQKRF”, “RRQRRF”, and “RRKKRF” cleavage sites, respectively. Interestingly, recent studies on amino acid composition of NDV F cleavage site revealed that strains with Q at the third position in the cleavage site are predicted to have an enhanced cell-cell spreading ability [27]. Thus, in future development of vaccines based on indigenous NDV isolates in Nigeria, special consideration should be given to those isolates with Q at the third position of their F cleavage site.

**Figure 1:**
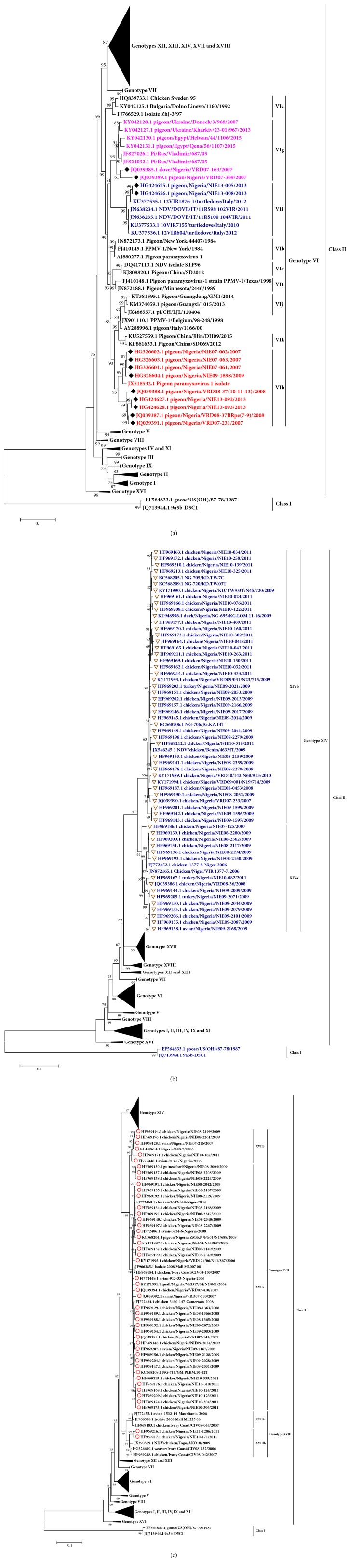
**Molecular phylogenetic analysis of complete F coding regions (1662bp) for Nigerian Newcastle disease virus isolates**. (a) Zoomed view of prevalent genotype VI isolates is shown. The coloured taxa indicate the subgenotypes with isolates prevalent in Nigeria (indicated with black icons at the node). (b) Relationship of Nigerian genotype XIV isolates with other reference strains is shown (all isolates prevalent in Nigeria are labelled with inverted triangle). (c) Expanded view of Nigerian genotype XVII and XVIII isolates (labelled with circles at the node). The evolutionary history was inferred using the maximum likelihood method based on the Tamura 3-parameter model. The tree with the highest log likelihood (-22231.3479) is shown. The percentage of trees in which the associated taxa clustered together is shown next to the branches. Initial tree(s) for the heuristic search were obtained by applying the neighbor-joining method to a matrix of pairwise distances estimated using the maximum composite likelihood (MCL) approach. A discrete Gamma distribution was used to model evolutionary rate differences among sites (5 categories (+*G*, parameter = 0.6931)). The tree is drawn to scale, with branch lengths measured in the number of substitutions per site. The analysis involved 195 nucleotide sequences. Codon positions included were 1st+2nd+3rd+Noncoding. All positions containing gaps and missing data were eliminated. Evolutionary analyses were conducted in MEGA6 [28].

Isolates of NDV belonging to the genotype VI group have been recovered from pigeons, doves, and chicken in the Northern (Kano and Jigawa) as well as the Southern (Oyo and Lagos) parts of Nigeria (Table 2). They are classified into subgenotypes VIg, VIh, and VIi with the overall average evolutionary divergence among the three subgenotypes being 7.3%. The highest genetic distance among these groups occurs between the subgenotypes VIh and VIi (Table 2). Surprisingly, the Nigerian genotype VIg isolates share a high degree of phylogenetic relationship with the Russian, Egyptian, and Ukrainian isolates whereas the genotype VIh isolates are more related to the pigeon paramyxovirus isolated from wild birds in Kenya (Figure 1(a)). On the other hand, the isolates grouped under subgenotypes VIi form the same phylogenetic cluster with the Italian strains. These close genetic relationships among the isolates could be of epidemiological significance and certainly suggest a recent common ancestry during their evolution [52, 53]. Given that these viruses can easily be transmitted from pigeons and doves to domesticated chicken especially at ecological contact surfaces [82–84] and that some of them have been shown to dramatically gain virulence upon a few passages in chicken [85, 86], their occurrence in Nigerian pigeon population economically threatens the poultry subsector in the country. Thus, there is a need to intensify disease surveillance in live birds markets, households, and commercial poultry farms, so that disease epidemics due to these isolates can be quickly detected and contained.

Isolates belonging to genotype XIV are the most predominantly isolated strains of NDV in Nigeria. Both subgenotypes, XIVa and XIVb, have been recovered from domestic birds found in the North-West (Sokoto, Kaduna, Jigawa), North-Central (Benue, Kogi), North-East (Taraba and Yobe), and South-Western parts of the country (Lagos) (Table 1). The intergroup genetic distance between the two subgenotypes averages at 6.7% (Table 3). Meanwhile, subgenotype XIVa isolates appear to be more genetically diverse, having an average intragenotype evolutionary divergence of 2.6%. On the other hand, isolates in the subgenotype XIVb are less divergent with about 98.6% overall mean similarity among themselves (data not shown). Phylogenetically, the genotype XIVa isolates form a cluster with some strains in Niger Republic while the Nigerian genotype XIVb isolates tend to be more closely related to the 2009 isolates from Benin Republic (Figure 1(b)). Interestingly, the isolates in genotype XIVa that share the highest nucleotide similarity with those from Niger Republic were all obtained from Sokoto State which shares a direct international border with Niger Republic. Their intimate phylogenetic relationship could therefore be partially explained by the cross border movements between the two countries which may facilitate the spread of the virus from one place to another. Notably, all genotype XIV isolates are so far restricted in distribution to only the West African subregion where they cause havoc in the regional poultry industry [11]. However, their emergence in other parts of the continent within the next few years would not be unexpected given the poor transboundary biosecurity measures in most of the African countries.

Several strains of NDV isolated in Nigeria from 2006-2011 belong to either subgenotype XVIIa or XVIIb, with the mean evolutionary divergence between the two subgenotypes being 4.1% (Table 3). Members of the subgenotypes XVIIa are highly similar, with an average nucleotide sequence similarity of 98.1% at the level of F protein gene. Surprisingly, despite their extensive spatial distribution in the northern states (Sokoto, Zamfara, Plateau, Gombe, and Yobe states), none of these isolates was recovered from the southern parts of the country. It is however not clear whether this is due to sampling bias or they truly do not exist in those areas. On the basis of phylogenetic analysis, genotype XVIIa isolates from Nigeria are closely related to those from Niger Republic, Cameroun, Burkina Faso, and Mali whereas the genotype XVIIb isolates, whose mean intrasubgenotype distance was estimated to be 1.5%, are so far exclusively composed of Nigerian strains (Figure 1(c)). Importantly, the ecological distribution of genotype XVII isolates is to date restricted to the West and Central Africa [32, 53] where they are believed to considerably militate against poultry production. Indeed, representatives of these isolates have recently been shown to cause a typical velogenic viscerotropic ND [87] characterized by end stage morbidity and high mortality in chicken. There is therefore need to intensify the ongoing passive and active surveillance for ND in various parts of the country in order to avert the potential economic losses due to outbreaks with these strains.

Two highly similar sequences (99%) obtained from Nigerian NDV strains in the NCBI database were categorised under the subgenotype XVIIIb. They were obtained from Sokoto State in the North and Oyo State from the South. Based on the phylogenetic tree analysis, the two strains are quite related to the isolates from Togo and Ivory-Coast (Figure 1(c)) as earlier reported by Shittu et al. [32]. On the contrary, subgenotype XVIIIa isolates are yet to be encountered in Nigeria. Surprisingly, the interpopulation evolutionary distance between the two isolates in the subgenotype XVIIIb and those in either subgenotype XVIIa or XVIIb is slightly lower than the 10% cut-off for differentiating new genotypes (Table 2). This discrepancy was earlier observed by [88] who wondered if genotype XVIII isolates could be another subgroup of genotype XVII. However, Snoeck and Muller (2016) maintained that the two genotypes (XVII and XVIII) still stand and that the parameter used by Desingu et al. to challenge the existence of genotype XVIII was incorrect. Therefore, it is possible that the slightly lower than the threshold interpopulation distance observed in this study was due to the small number of genotype XVIII sequences from Nigeria (n=2) used in the analysis. As all the known genotype XVIII isolates are predicted to be virulent in chicken, their emergence in other parts of the country should be carefully monitored as part of the usual disease surveillance programme in the country.

## 4. Challenges for Newcastle Disease Control in Nigeria

Vaccination remains the most practical method of disease control in poultry and therefore plays a major role in strengthening the modern poultry industry [89, 90]. The ultimate goal of any vaccination program is the induction of sterilising immunity in the vaccinated host [91]. However, this is hardly achievable in poultry [89], owing to numerous factors that may adversely affect the efficacy of vaccination. The fact that all NDV strains are grouped into one serotype [92] suggests that immunity developed against one strain should offer cross protection against challenge with any other strains. Unfortunately to date, outbreaks of ND are frequently reported among farms that have vaccinated using the available vaccines [32, 52, 53]. The cause of these disease outbreaks among the vaccinated birds is still controversial in the literature. While some researchers hold the view that the poor vaccine induced immunity is due to the suboptimal vaccine intake following its mass administration in poultry [93], others believe that the genetic variation between the vaccine and the circulating field strains might be the major factor responsible for the incomplete protective efficacy of the current vaccines [94, 95]. Although the currently used vaccines, when correctly administered, are known to fully protect birds against clinical disease and mortality [95, 96], they cannot block the replication of the virulent virus post challenge [44, 45]. Thus, the vaccinated birds may look apparently healthy but still excrete a large amount of the virulent virus, which can in turn cause disease among unprotected birds. Since it is an established fact that ND vaccines are more effective in reducing virus shedding when the vaccine strains are genetically closer to the challenge strain [57, 97], the evolutionary distance between the vaccine strains and the circulating field strain represents an important factor in effective disease control, since it explains the continuous occurrence of ND outbreaks despite the extensive poultry vaccination programs in the country.

Based on the evolutionary analysis of the complete F coding sequences performed in this study, all the virulent NDV genotypes circulating in Nigeria are shown to be distantly related to the currently available vaccine strains in the country (Table 4). LaSota which is the most widely used live attenuated ND vaccine in Nigeria and indeed many parts of the world has an average nucleotide sequence divergence of 15.7-18.6% when compared with all the existing virulent class II subgenotypes in Nigeria (Table 4). Similarly, the very popular Komarov inactivated NDV vaccine differs from the circulating NDV subgenotypes in the country with an average evolutionary distance of 15.5-18% (Table 4). Recently in Indonesia, sequence divergence between the field and the vaccine strains has been implicated in a severe disease outbreak that led to 70% mortality among the vaccinated birds [95]. Furthermore, in Malaysia where the prevalent isolates are genotype VII strains that considerably diverge from the LaSota strain, the frequency of ND outbreaks among the vaccinated farms has steadily increased from 2009 to date [62, 98]. Therefore, the wide genetic divergence between the Nigerian NDV strains and vaccine strains used in the country should be a source of a serious concern to the national poultry industry and requires urgent attention. These problems collectively highlight the possible limitations of the current vaccines in offering a complete protection against the circulating strains of NDV in Nigeria. The need to improve the current disease control strategies is therefore imperative.

## 5. Way Forward

The panacea for all these ND control challenges in Nigeria is the maintenance of strict biosecurity and the development of rationally designed vaccines based on the currently circulating isolates in the country. With the advent of reverse genetics technology that allows the recovery of recombinant NDV from their cloned cDNA [99], genotype-matched live attenuated vaccines can be easily generated. Since the complete genome sequence of some biologically well-characterized viruses in the country has already been obtained [33, 34], efforts should be intensified towards rescuing their attenuated counterparts by simply engineering their F cleavage site to encode monobasic amino acid residues instead of the poly basic motifs [100]. By developing a reverse genetics system for one prevalent strain in the country, vaccine candidates against all the circulating strains can easily be obtained by F gene swapping in the full length infectious clone followed by the recovery of the chimeric viruses by reverse genetics techniques. Alternatively, recombinant viral vectors such as herpesvirus of turkey [101] (HVT) expressing surface glycoproteins (F and/or HN) of the circulating NDV can be developed as an effective genotype-matched vaccine against the prevailing genotypes in the country.

## 6. Conclusion

In summary, a comprehensive distribution of NDV genotypes in various regions of Nigeria has been provided. Apparently, multiple genetically distinct strains of NDV are cocirculating in some states of the federation, an important factor that may favour the emergence of novel virulent isolates in the country. In particular, apart from genotype VI isolates, all the virulent NDV genotypes prevalent in Nigeria have been isolated in Sokoto State between 2007 and 2011, making the State a potential hotspot of different NDV genotypes in Nigeria. It is interesting to know that genotype VII isolates responsible for the on-going fourth and the imminent fifth ND panzootic [102, 103] have not been reported in Nigeria despite their recent emergence in some African countries [104, 105]. Since these panzootic viruses have a high potential for international spread, there is a need to intensify disease surveillance activities and strengthen biosecurity barriers so as to avoid their introduction into the country. Finally, given the wide evolutionary divergence between the commonly used vaccines and the circulating NDV strains in the country, there is a need to revise the current ND control strategies in Nigeria. Genotype-matched vaccines with improved protective efficacy and virus shedding blocking ability should be designed to specifically target the currently circulating NDV genotypes in the country.

## Figures and Tables

**Table 1:** Current classification and distribution of class II NDV genotypes.

**Genotypes**	**Subgenotypes**	**Geographic distribution**	**Remarks**
**I**	Ia, Ib, Ic	Australia, Africa, Europe, US, Asia	Low virulence, Ulster, V4
**II**	-	North and South America, Africa, Asia and Europe	Avirulent, lentogenic, Lasota, B1
**III**	-	Japan and Australia, Taiwan, Zimbabwe	Ancient strains but still emerging, mesogenic Mukteshwar
**IV**	-	Europe, Africa, Asia	Virulent, Herts/33 (UK)
**V**	Va, Vb, Vc, Vd	South America, Europe and Africa	Virulent, Anhinga (US)
**VI**	VIa, VIb, VIc, VId, VIe, VIf, VIg, VIh, VIi, VIj, VIk	Europe, Asia, Africa, South America	Pigeon paramyxoviruses
**VII**	VIIa, VIIb, VIIc,, VIId, VIIe, VIIf, VIIg, VIIh, VIIi	Emerged in Far East in 1990, spread to Europe and Asia, Africa.	Virulent, 4th ND panzootic virus, 5th panzootic virus
**VIII**	-	South Africa, Asia	Highly virulent, AF22440
**IX**	-	First isolated in China in 1948	Highly virulent
**X**	-	Taiwan, Argentina, USA	Virulent
**XI**	-	Madagascar	Virulent, restricted distribution
**XII**	-	South America and China	Virulent
**XIII**	XIIIa, XIIIb, XIIIc	Asia, Europe and Africa	Virulent, continuously emerging
**XIV**	XIVa, XIVb	West Africa	Highly virulent, recovered from domestic birds only
**XV**	-	China	Originated from mixed virulent and vaccine viruses
**XVI**	-	Europe in 1940s, Africa and Asia in 1980s	Highly related to genotype IV
**XVII**	XVIIa, XVIIb	West and Central Africa	Highly virulent, continuously emerging evolving
**XVIII**	XVIIIa, XVIIIb	West Africa	Highly virulent

**Table 2:** Some features of the Newcastle disease virus subgenotypes found in Nigeria.

**Subgenotype**	**Strain Identity**	**Year of isolation**	**Cleavage site**	**Location**
VIg	JQ039385.1 dove/Nigeria/VRD07-163/2007	2007	RRQKRF	Kano
	JQ039389.1 pigeon/Nigeria/VRD07-369/2007	2007	KRQKRF	Jigawa

VIh	HG326601.1 pigeon/Nigeria/NIE07-061/2007	2007	RRKKRF	Oyo
	HG326602.1 pigeon/Nigeria/NIE07-062/2007	2007	RRKKRF	Oyo
	HG326603.1 pigeon/Nigeria/NIE07-063/2007	2007	RRKKRF	Oyo
	HG326604.1 pigeon/Nigeria/NIE09-1898/2009	2009	RRKKRF	Lagos
	HG424627.1 pigeon/Nigeria/NIE13-092/2013	2013	RRRKRF	Oyo
	HG424628.1 pigeon/Nigeria/NIE13-093/2013	2013	RRRKRF	Oyo
	JQ039387.1 Nigeria/VRD08-37BRpe(7-9)/2008	2008	RRRKRF	Jigawa
	JQ039388.1 Nigeria/VRD08-37(10-11-13)/2008	2007	RRRKRF	Jigawa
	JQ039391.1 Nigeria/VRD07-231/2007	2007	RRRKRF	Jigawa

VIi	HG424625.1 pigeon/Nigeria/NIE13-005/2013	2013	RRQKRF	Oyo
	HG424626.1 pigeon/Nigeria/NIE13-008/2013	2013	RRQKRF	Oyo

XIVa	HF969131.1 chicken/Nigeria/NIE08-2117/2009	2009	RRQKRF	Sokoto
	HF969136.1 chicken/Nigeria/NIE08-2194/2009	2009	RRQKRF	Sokoto
	HF969139.1 chicken/Nigeria/NIE08-2280/2009	2009	RRQKRF	Sokoto
	HF969144.1 chicken/Nigeria/NIE09-2009/2009	2009	RRQKRF	Yobe
	HF969150.1 chicken/Nigeria/NIE09-2044/2009	2009	RRQKRF	Yobe
	HF969153.1 chicken/Nigeria/NIE09-2079/2009	2009	RRQKRF	Yobe
	HF969155.1 chicken/Nigeria/NIE09-2087/2009	2009	RRQKRF	Yobe
	HF969167.1 turkey/Nigeria/NIE10-082/2011	2011	RRQKRF	Sokoto
	HF969186.1 chicken/Nigeria/NIE07-125/2007	2007	RRQKRF	Lagos
	HF969193.1 chicken/Nigeria/NIE08-2150/2009	2009	RRQKRF	Sokoto
	HF969200.1 chicken/Nigeria/NIE08-2362/2009	2009	RRQKRF	Sokoto
	HF969205.1 turkey/Nigeria/NIE09-2071/2009	2009	RRQKRF	Yobe
	HF969206.1 chicken/Nigeria/NIE09-2101/2009	2009	RRQKRF	Yobe
	JQ039386.1 VRD08-36/2008	2008	RRQKRF	Taraba
	HF969158.1 avian/Nigeria/NIE09-2168/2009	2009	RRQKRF	Yobe

XIVb	HF969133.1 chicken/Nigeria/NIE08-2159/2009	2009	RRRKRF	Sokoto
	HF969141.1 chicken/Nigeria/NIE08-2359/2009	2009	RRRKRF	Sokoto
	HF969142.1 chicken/Nigeria/NIE09-1596/2009	2009	RRQKRF	Benue
	HF969143.1 chicken/Nigeria/NIE09-1597/2009	2009	RRQKRF	Benue
	HF969145.1 chicken/Nigeria/NIE09-2014/2009	2009	RRRKRF	Yobe
	HF969146.1 chicken/Nigeria/NIE09-2017/2009	2009	RRRKRF	Yobe
	HF969149.1 chicken/Nigeria/NIE09-2041/2009	2009	RRRKRF	Yobe
	HF969151.1 chicken/Nigeria/NIE09-2053/2009	2009	RRRKRF	Yobe
	HF969157.1 chicken/Nigeria/NIE09-2166/2009	2009	RRRKRF	Yobe
	HF969161.1 chicken/Nigeria/NIE10-024/2011	2011	RRRKRF	Sokoto
	HF969162.1 chicken/Nigeria/NIE10-032/2011	2011	RRRKRF	Sokoto
	HF969163.1 chicken/Nigeria/NIE10-034/2011	2011	RRQKRF	Sokoto
	HF969164.1 chicken/Nigeria/NIE10-041/2011	2011	RRRKRF	Sokoto
	HF969165.1 chicken/Nigeria/NIE10-043/2011	2011	RRRKRF	Sokoto
	HF969166.1 chicken/Nigeria/NIE10-076/2011	2011	RRRKRF	Sokoto
	HF969169.1 chicken/Nigeria/NIE10-150/2011	2011	RRRKRF	Sokoto
	HF969170.1 chicken/Nigeria/NIE10-160/2011	2011	RRRKRF	Sokoto
	HF969172.1 chicken/Nigeria/NIE10-258/2011	2011	RRQKRF	Sokoto
	HF969173.1 chicken/Nigeria/NIE10-302/2011	2011	RRRKRF	Sokoto
	HF969177.1 chicken/Nigeria/NIE10-409/2011	2011	RRRKRF	Sokoto
	HF969178.1 chicken/Nigeria/NIE08-2270/2009	2009	RRRKRF	Sokoto
	HF969187.1 chicken/Nigeria/NIE08-0453/2008	2008	RRRKRF	Yobe
	HF969190.1 chicken/Nigeria/NIE08-2032/2009	2009	RRRKRF	Sokoto
	HF969198.1 chicken/Nigeria/NIE08-2279/2009	2009	RRRKRF	Yobe
	HF969201.1 chicken/Nigeria/NIE09-1599/2009	2009	RRQKRF	Benue
	HF969202.1 chicken/Nigeria/NIE09-2013/2009	2009	RRRKRF	Yobe
	HF969203.1 turkey/Nigeria/NIE09-2021/2009	2009	RRRKRF	Yobe
	HF969208.1 chicken/Nigeria/NIE10-122/2011	2011	RRRKRF	Sokoto
	HF969210.1 chicken/Nigeria/NIE10-139/2011	2011	RRQKRF	Sokoto
	HF969211.1 chicken/Nigeria/NIE10-263/2011	2011	RRRKRF	Sokoto
	HF969212.1 chicken/Nigeria/NIE10-318/2011	2011	RRRKRF	Sokoto
	HF969213.1 chicken/Nigeria/NIE10-325/2011	2011	RRQKRF	Sokoto
	HF969214.1 chicken/Nigeria/NIE10-333/2011	2011	RRRKRF	Sokoto
	JQ039390.1 chicken/Nigeria/VRD07-233/2007	2007	RRRKRF	-
	KC568205.1 NG-705/KD.TW.7C	2009	RRQKRF	Kaduna
	KC568206.1 NG-706/JG.KZ.14T	2009	RRRKRF	Jigawa
	KC568209.1 NG-720/KD.TW.03T	2009	RRQKRF	Kaduna
	KT948996.1 duck/Nigeria/NG-695/KG.LOM.11-16/2009	2009	RRRKRF	Kogi
	KY171989.1 chicken/Nigeria/VRD10/143/N68/913/2010	2010	RRRKRF	-
	KY171990.1 chicken/Nigeria/KD/TW/03T/N45/720/2009	2009	RRQKRF	Kaduna
	KY171993.1 chicken/Nigeria/VRD09/031/N23/715/2009	2009	RRRKRF	-
	KY171994.1 chicken/Nigeria/VRD09/001/N19/714/2009	2009	RRRKRF	-

Unassigned	KC568207.1 NG-707/GM.GMM.17-18T	2009	RRRKRF	Gombe
	KU058680.1 duck/Nigeria/903/KUDU-113/1992	1992	RRQKRF	-

XVIIa	FJ772449.1 avian-913-33-Nigeria-2006	2006	RRQKRF	-
	FJ772486.1 avian-3724-6-Nigeria-2008	2008	RRQKRF	-
	HF969129.1 chicken/Nigeria/NIE08-1363/2008	2008	RRQKRF	Plateau
	HF969130.1 guinea fowl/Nigeria/NIE08-2004/2009	2009	RRQKRF	Sokoto
	HF969132.1 chicken/Nigeria/NIE08-2149/2009	2009	RRQKRF	Sokoto
	HF969134.1 chicken/Nigeria/NIE08-2168/2009	2009	RRQKRF	Sokoto
	HF969135.1 chicken/Nigeria/NIE08-2187/2009	2009	RRQKRF	Sokoto
	HF969137.1 chicken/Nigeria/NIE08-2208/2009	2009	RRQKRF	Sokoto
	HF969138.1 chicken/Nigeria/NIE08-2224/2009	2009	RRQKRF	Sokoto
	HF969140.1 chicken/Nigeria/NIE08-2340/2009	2009	RRQKRF	Sokoto
	HF969147.1 chicken/Nigeria/NIE09-2031/2009	2009	RRQKRF	Yobe
	HF969148.1 chicken/Nigeria/NIE09-2034/2009	2009	RRQKRF	Yobe
	HF969152.1 chicken/Nigeria/NIE09-2072/2009	2009	RRQKRF	Yobe
	HF969154.1 chicken/Nigeria/NIE09-2083/2009	2009	RRQKRF	Yobe
	HF969156.1 chicken/Nigeria/NIE09-2128/2009	2009	RRQKRF	Yobe
	HF969168.1 chicken/Nigeria/NIE10-124/2011	2011	RRRKRF	Sokoto
	HF969174.1 chicken/Nigeria/NIE10-304/2011	2011	RRRKRF	Sokoto
	HF969175.1 chicken/Nigeria/NIE10-306/2011	2011	RRRKRF	Sokoto
	HF969176.1 chicken/Nigeria/NIE10-310/2011	2011	RRRKRF	Sokoto
	HF969188.1 chicken/Nigeria/NIE08-1365/2008	2008	RRQKRF	Plateau
	HF969189.1 chicken/Nigeria/NIE08-1366/2008	2008	RRQKRF	Plateau
	HF969191.1 chicken/Nigeria/NIE08-2042/2009	2009	RRQKRF	Sokoto
	HF969192.1 chicken/Nigeria/NIE08-2119/2009	2009	RRQKRF	Sokoto
	HF969195.1 chicken/Nigeria/NIE08-2247/2009	2009	RRQKRF	Sokoto
	HF969197.1 chicken/Nigeria/NIE08-2267/2009	2009	RRQKRF	Sokoto
	HF969199.1 chicken/Nigeria/NIE08-2349/2009	2009	RRQKRF	Sokoto
	HF969204.1 chicken/Nigeria/NIE09-2028/2009	2009	RRQKRF	Yobe
	HF969207.1 avian/Nigeria/NIE09-2167/2009	2009	RRQKRF	Yobe
	HF969209.1 chicken/Nigeria/NIE10-123/2011	2011	RRRKRF	Sokoto
	HF969215.1 chicken/Nigeria/NIE10-335/2011	2011	RRRKRF	Sokoto
	JQ039392.1 avian/Nigeria/VRD07-733/2007	2007	RRQKRF	-
	JQ039393.1 chicken/Nigeria/VRD07-141/2007	2007	RRQKRF	Sokoto
	JQ039394.1 chicken/Nigeria/VRD07-410/2007	2007	RRQKRF	Jigawa
	KC568204.1 pigeon/Nigeria/ZM/KN/PG01/N1/688/2009	2009	RRQKRF	Zamfara
	KC568208.1 NG-710/GM.PLBM.10-12T	2009	RRQKRF	Gombe
	KY171991.1 Nigeria/VRD17/04/N2/861/2004	2004	RRQKRF	-
	KY171992.1 chicken/Nigeria/JN/469/N44/892/2009	2009	RRQKRF	Plateau
	KY171995.1 chicken/Nigeria/VRD124/06/N11/867/2006	2006	RRQKRF	-

XVIIb	FJ772446.1 avian-913-1-Nigeria-2006	2006	RRQKRF	-
	HF969128.1 avian/Nigeria/NIE07-216/2007	2007	RRQKRF	-
	HF969171.1 chicken/Nigeria/NIE10-182/2011	2011	RRQKRF	Sokoto
	HF969194.1 chicken/Nigeria/NIE08-2199/2009	2009	RRQKRF	Sokoto
	HF969196.1 chicken/Nigeria/NIE08-2261/2009	2009	RRQKRF	Sokoto
	KF442614.1 Nigeria/228-7/2006	2006	RRQRRF	-

XVIIIb	HF969216.1 chicken/Nigeria/NIE11-1286/2011	2011	RRQKRF	Oyo
	HF969217.1 chicken/Nigeria/NIE10-171/2011	2011	RRQKRF	Sokoto

Class I	HG326605.1 Spur-winged goose/Nigeria/ NIE-08-0121/2008	2008	GKQGRL	Yobe
	HG326606.1 Spur-winged goose/Nigeria/ NIE-08-0121/2008	2008	GKQGRL	Yobe
	HG326607.1 Spur-winged goose/Nigeria/ NIE-08-0121/2008	2008	GKQGRL	Yobe
	HG326608.1 Spur-winged goose/Nigeria/ NIE-08-0121/2008	2008	GKQGRL	Yobe

**Table 3:** Estimates of evolutionary divergence among the virulent NDV genotypes circulating in Nigeria.

	**VIg **	**VIh**	**VIi**	**XIVa**	**XIVb**	**XVIIa**	**XVIIb**	**XVIIIb**
**VIg **		(0.005)	(0.006)	(0.007)	(0.008)	(0.007)	(0.007)	(0.008)
**VIh**	0.081		(0.007)	(0.007)	(0.007)	(0.007)	(0.007)	(0.007)
**VIi**	0.099	0.113		(0.008)	(0.009)	(0.008)	(0.008)	(0.008)
**XIVa**	0.127	0.128	0.149		(0.004)	(0.005)	(0.006)	(0.006)
**XIVb**	0.132	0.135	0.146	0.067		(0.007)	(0.007)	(0.007)
**XVIIa**	0.124	0.128	0.136	0.106	0.109		(0.004)	(0.006)
**XVIIb**	0.122	0.126	0.135	0.104	0.105	0.041		(0.007)
**XVIIIb**	0.118	0.120	0.132	0.112	0.114	0.093	0.095	

The table shows number of base differences per site from averaging over all sequence pairs between groups. Standard error estimate(s) are shown above the diagonal and were obtained by a bootstrap procedure (500 replicates). The analysis involved 120 nucleotide sequences. Codon positions included were 1st+2nd+3rd+Noncoding. All positions containing gaps and missing data were eliminated. Evolutionary analyses were conducted in MEGA6 [28].

**Table 4:** Genetic distances between the common vaccine strains and the prevalent virulent NDV subgenotypes in Nigeria.

**Vaccine**	**Prevalent subgenotype**							
	** VIg**	** VIh**	** VIi**	** XIVa**	** XIVb**	** XVIIa**	** XVIIb**	**XVIIIb**
**B1**	0.156	0.155	0.162	0.173	0.185	0.165	0.173	0.16
**Komarov**	0.155	0.155	0.161	0.169	0.180	0.157	0.165	0.159
**Lasota**	0.159	0.157	0.166	0.174	0.186	0.165	0.173	0.162
**V4**	0.147	0.151	0.156	0.165	0.175	0.153	0.16	0.152
**VGGA**	0.162	0.162	0.169	0.179	0.191	0.17	0.178	0.166
**I2**	0.145	0.153	0.155	0.163	0.173	0.151	0.149	0.156

The table shows number of base substitutions per site of the complete F gene sequence pairs between vaccine strains and NDV subgenotypes in Nigeria. The analysis involved 126 nucleotide sequences. Codon positions included were 1st+2nd+3rd+Noncoding. All positions containing gaps and missing data were eliminated. Evolutionary analyses were conducted in MEGA6 [28].
